# Advanced Thermal Imaging Processing and Deep Learning Integration for Enhanced Defect Detection in Carbon Fiber-Reinforced Polymer Laminates

**DOI:** 10.3390/ma18071448

**Published:** 2025-03-25

**Authors:** Renan Garcia Rosa, Bruno Pereira Barella, Iago Garcia Vargas, José Ricardo Tarpani, Hans-Georg Herrmann, Henrique Fernandes

**Affiliations:** 1 Faculty of Computing, Federal University of Uberlandia, Uberlandia 38408-100, Brazil; renan.garcia@ufu.br (R.G.R.); brunobarella@ufu.br (B.P.B.); iagogarcia@ufu.br (I.G.V.); 2 Department of Materials, Sao Carlos School of Engineering, University of Sao Paulo, Sao Carlos 13566-590, Brazil; jrpan@sc.usp.br; 3 Fraunhofer IZFP Institute for Non-Destructive Testing, Campus E3 1, 66123 Saarbrucken, Germany; hans-georg.herrmann@izfp.fraunhofer.de; 4 Chair for Lightweight Systems, Saarland University, Campus E3 1, 66123 Saarbrucken, Germany; 5 IVHM Centre, Faculty of Engineering and Applied Sciences, Cranfield University, Cranfield MK43 0AL, UK

**Keywords:** pulsed thermography, carbon fiber-reinforced polymer, thermal image preprocessing, non-destructive testing (NDT), deep learning, polynomial approximation

## Abstract

Carbon fiber-reinforced polymer (CFRP) laminates are widely used in aerospace, automotive, and infrastructure industries due to their high strength-to-weight ratio. However, defect detection in CFRP remains challenging, particularly in low signal-to-noise ratio (SNR) conditions. Conventional segmentation methods often struggle with noise interference and signal variations, leading to reduced detection accuracy. In this study, we evaluate the impact of thermal image preprocessing on improving defect segmentation in CFRP laminates inspected via pulsed thermography. Polynomial approximations and first- and second-order derivatives were applied to refine thermographic signals, enhancing defect visibility and SNR. The U-Net architecture was used to assess segmentation performance on datasets with and without preprocessing. The results demonstrated that preprocessing significantly improved defect detection, achieving an Intersection over Union (IoU) of 95% and an F1-Score of 99%, outperforming approaches without preprocessing. These findings emphasize the importance of preprocessing in enhancing segmentation accuracy and reliability, highlighting its potential for advancing non-destructive testing techniques across various industries.

## 1. Introduction

Carbon fiber-reinforced polymers (CFRPs) have revolutionized industries where lightweight and high-strength materials are essential, such as aerospace, automotive, and renewable energy sectors [1]. Their exceptional combination of mechanical and physical properties has made them indispensable in applications demanding high-performance solutions. However, the structural integrity of CFRPs can be compromised during manufacturing or in-service use due to defects such as delaminations, cracks, and voids, posing significant safety and reliability risks [1]. The formation and progression of these defects are influenced by various factors, including manufacturing inconsistencies and prolonged exposure to harsh environmental conditions. High temperatures, corrosive agents, and mechanical stresses (both dynamic and static) can degrade the resin matrix and weaken the fiber–resin interface, leading to structural failures over time [2]. In addition, sustained loading combined with temperature cycling has been shown to significantly weaken CFRP bonds, accelerating failure. Exposure to saline environments further exacerbates degradation through galvanic corrosion, particularly when CFRP is in direct contact with steel. Moisture ingress can plasticize adhesives and lower bond strength, while fatigue loading can cause progressive debonding at CFRP–steel interfaces. Studies have indicated that CFRP systems subjected to long-term environmental exposure can lose up to 60% of their original bond strength [3]. Understanding these degradation mechanisms is crucial for improving both defect detection and preventive maintenance strategies.

Non-destructive testing (NDT) techniques play a pivotal role in detecting internal defects without compromising the functionality of the tested components. Several NDT methods have been employed to inspect CFRP laminates, each with distinct advantages and limitations. Ultrasonic testing (UT) is widely used due to its sensitivity to subsurface defects. Yet, it faces challenges when inspecting highly attenuative composite materials [4]. X-ray computed tomography (XCT) provides detailed internal imaging but is time-consuming and costly, limiting its practical application in large-scale inspections [5]. Eddy current testing (ECT) is effective for conductive composites but has limited applicability to non-conductive CFRPs. In this context, pulsed thermography (PT) has emerged as a preferred method due to its ability to provide rapid and comprehensive assessments of composite materials’ structural health [5]. PT operates by applying a heat pulse to the material’s surface and analyzing the thermal response, allowing defect detection based on variations in heat diffusion properties [6]. It offers a non-invasive and efficient solution for defect detection. Despite its advantages, raw thermographic data often exhibit substantial noise and thermal variations, complicating accurate defect identification [7]. Consequently, there is a growing need for advanced preprocessing methods to enhance the quality and interpretability of thermographic data.

A recent study demonstrated the effectiveness of combining self-organizing maps (SOMs) with bio-inspired parameter optimization through bee colony optimization (BCO) techniques in improving thermographic image analysis [8]. However, these methods often require extensive computational resources and can lack generalizability across different CFRP structures and defect types, limiting their broader adoption [9]. This methodology highlights the potential of advanced algorithms in improving non-destructive testing workflows and supports the exploration of similar innovations in the field.

Thermographic Signal Reconstruction (TSR) has been proposed as a robust preprocessing technique to improve the signal-to-noise ratio (SNR) in thermographic images [7]. By employing polynomial fitting and derivative analysis, TSR refines the thermal signals, enabling clearer visualization of defect features. Nonetheless, one of the primary challenges in thermographic image processing lies in balancing noise reduction while preserving critical defect-related thermal signatures. Over-smoothing can obscure defect boundaries, whereas inadequate filtering may fail to suppress noise effectively [10]. With the rise of deep learning methods, particularly convolutional neural networks (CNNs) such as U-Net, the ability to preprocess data effectively has become even more critical [11,12].

Deep learning models like U-Net have shown significant promise in the segmentation of thermographic images, achieving high accuracy in identifying complex patterns and defects [13]. However, the performance of these networks heavily depends on the quality of input data. Despite advancements in PT and deep learning, studies explicitly quantifying the impact of preprocessing methods like TSR on U-Net performance remain scarce. While prior research has successfully applied deep learning techniques to defect detection, the direct influence of preprocessing on segmentation accuracy and model robustness has not been fully addressed. A systematic investigation of how preprocessing steps enhance deep learning-based defect segmentation is still lacking in the literature. Addressing this gap is crucial to advancing the integration of NDT with artificial intelligence for composite materials.

This work aims to systematically investigate the influence of TSR preprocessing on the performance of the U-Net architecture in detecting and segmenting defects in CFRPs. By comparing the segmentation results of U-Net models trained on raw and TSR-preprocessed thermographic data, we seek to demonstrate how preprocessing can significantly enhance defect detection capabilities. Metrics such as F1-Score and Intersection over Union (IoU) will be employed to evaluate the effectiveness of the proposed approach. In comparison to existing studies, this research uniquely quantifies the impact of TSR preprocessing on deep learning-driven defect segmentation, providing a structured analysis of its benefits and trade-offs. By bridging the gap between thermographic preprocessing and deep learning-based defect detection, this study offers new insights into optimizing defect identification pipelines for high-performance composite materials.

## 2. Materials and Methods

**Figure 1:**
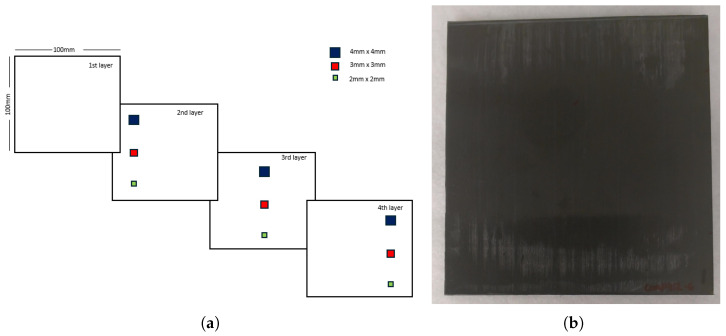
(**a**) Positions and sizes of defects in the laminate layers. (**b**) Inspected sample.

This study focuses on a unidirectional carbon fiber-reinforced polymer (CFRP) laminate composed of carbon/PEEK (polyether ether ketone) APC-2/AS4, a material known for its high strength-to-weight ratio, making it widely used in aerospace structural applications. CFRP structures are susceptible to defects such as delaminations and inclusions, which can compromise their mechanical performance and pose risks of operational failure. To simulate these defects, polyimide film inserts with a thickness (Kapton^®^ from 3M, St. Paul, MN, USA) were embedded in the laminate during manufacturing. These inserts had dimensions of 4 × 4 mm, 3 × 3 mm, and 2 × 2 mm and were placed at specific depths to replicate internal anomalies, as shown in Figure 1.

The laminate sample used in this experiment consisted of a flat CFRP panel with nine artificial defects. The laminate was manufactured using APC-2/AS4 (© Syensqo, Brussels, Belgium), which contains a fiber volume fraction of 61% and a ${\left[0_{2}/90_{2}\right]}_{6}$ stacking sequence. The defect positions within the laminate are illustrated in Figure 1, with nominal depths of 0.13 mm (D1), 0.26 mm (D2), and 0.39 mm (D3). The thermal properties of APC-2/AS4 are presented in Table 1 while mechanical properties are presented in Table 2.

**Figure 2:**
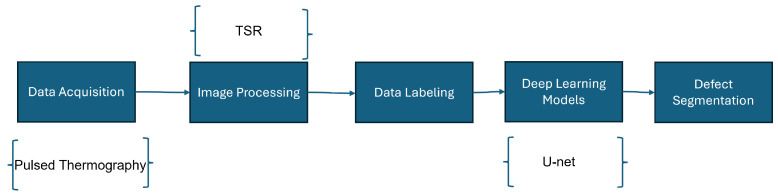
Defect segmentation methodology.

The approach employed in this study involves a four-phase process, outlined in Figure 2: capturing thermographic images via pulsed thermography, enhancing data quality through Thermographic Signal Reconstruction (TSR), annotating defects manually (process detailed in Section 2.5), and utilizing neural networks for defect segmentation. This methodology facilitates a thorough investigation of how TSR preprocessing affects the performance of deep learning models.

### 2.1. Pulsed Infrared Thermography

**Figure 3:**
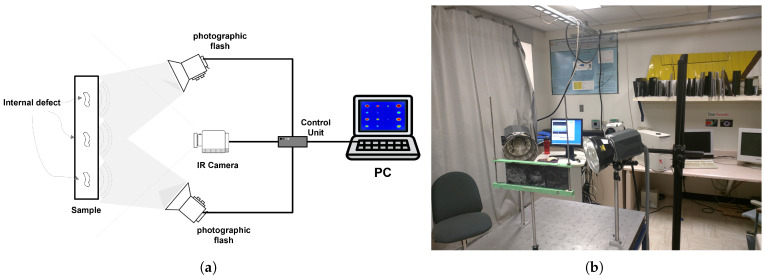
(**a**) Schematic of the experimental setup for pulsed thermography inspection. (**b**) Photograph of the work’s experimental setup.

Pulsed thermography is an active infrared non-destructive testing (NDT) technique that utilizes thermodynamic principles and infrared imaging to assess material properties and identify internal defects. This method involves applying a short-duration heat pulse to the material’s surface, resulting in a localized temperature rise. The heat propagates through the material, and internal discontinuities, such as delaminations and inclusions, alter the thermal diffusion, causing localized variations in the thermal response. The material’s thermal behavior is recorded over time using an infrared camera, capturing the emitted radiation. By analyzing the cooling process, defects can be identified based on temperature contrast, as these anomalies create variations in the thermal response [16,17]. Figure 3 illustrates the setup used in a typical pulsed thermography inspection.

Pulsed thermography has been used for years, and its main advantages and disadvantages are as follows [10]:

Advantages

- Fast surface inspection: While traditional ultrasound inspection can take several minutes (even hours), a thermographic inspection takes only a few seconds.
- Ease of installation: In many cases, no special preparation is required for the inspection. Only an infrared camera and a heat source are sufficient. Additionally, no contact with the surface is necessary.
- Safety: Unlike X-ray inspections, no harmful waves are used. However, stimulation using strong heat sources (such as photographic flashes) requires eye protection, and mechanical wave-generated heat requires ear protection.
- Inspection requires access to only one surface: Often, the inspected piece is installed and in use, making it impossible to move. Infrared thermography allows for on-site inspection with access to just one surface.
- Easy numerical thermal modeling: A numerical model of a thermographic experiment typically involves only heat transfer in solids, which can be easily solved using the finite element method and simulation software.
- Easy interpretation of thermograms: Unlike ultrasound inspection, where results are often 1D waveforms, infrared thermography provides 2D images, making it easier to distinguish defective and non-defective areas.
- Wide range of applications: Infrared thermography is used in various fields, including
  - Monitoring and diagnostics of electrical components, thermal comfort, buildings, and artwork.
  - Process control, such as sealing line inspection of Tetra Pak^®^ packaging (Meyrin, Switzerland), automotive brake system efficiency, and heat loss in electronic modules.
  - Detection of discontinuities, metal corrosion, cracks, and impact damage.
  - Material characterization, including thermal properties, moisture content, and fiber orientation.

Disadvantages

- Different emissivity of inspected materials: Low-emissivity materials reflect a lot of ambient thermal radiation, which can interfere with inspections. When possible, surface painting with spray can adjust emissivity for better results.
- Thermal losses: Heat loss due to conduction and radiation may lead to misinterpretation of results.
- Equipment cost: Infrared cameras and thermal stimulation units used in active thermography are more expensive than some other non-destructive testing techniques (e.g., visual inspection and basic ultrasound equipment). However, costs are competitive when compared to advanced technologies like phased arrays (ultrasound and eddy currents) and X-ray systems.
- Limited to detecting defects that alter thermal properties: Only defects that cause measurable thermal property changes can be detected.
- Reduced inspection depth: Infrared thermography is limited to a certain depth below the material surface. However, defects a few centimeters beneath the surface can be detected using low-frequency excitation in modulated thermography.
- Difficulty in achieving uniform heating: Achieving uniform heating, especially with photographic flashes, can be challenging.
- Transient nature of inspections: The transient thermal contrast requires infrared cameras capable of capturing sequential images.
- Need for a clear line of sight: The inspected object must be visible to the infrared camera without obstructions; otherwise, the inspection cannot be performed.

The following equation can mathematically describe the thermal behavior during pulsed thermography:

(1) $$y\left(z,t\right)=T_{0}+\frac{Q}{e\sqrt{\pi t}}\exp\left(-\frac{z^{2}}{4\alpha t}\right),$$

where α is the thermal diffusivity of the material, *k* is the thermal conductivity, $c_{p}$ is the heat capacity, $e=\sqrt{k\rho c_{p}}$ is the thermal effusivity, *z* represents the depth of the defect, and *t* is time.

At the material’s surface (z=0), the equation simplifies to

(2) $$y\left(t\right)=T_{0}+\frac{Q}{e\sqrt{\pi t}},$$

where $T_{0}$ is the initial temperature and *Q* is the applied heat flux [18].

The parameter *e* describes the material’s ability to exchange thermal energy with its surroundings. In a sound region with homogeneous material properties, thermal diffusivity and conductivity are consistent. However, defective regions exhibit distinct thermal behaviors due to different parameters. This temperature variation is what allows PT to successfully differentiate between sound and defective areas, serving as the foundation for non-destructive evaluation [16].

Additionally, the thermal effusivity *e* can be calculated as

(3) $$e=\sqrt{k\rho c_{p}},$$

where ρ represents the material’s density. This parameter plays a crucial role in heat transfer analysis and is fundamental in interpreting thermographic data for defect detection [17].

For the experimental setup, a high-power optical heating system was used to generate the thermal pulse. The heat source was positioned at a controlled distance from the CFRP surface to ensure uniform heat distribution. An infrared camera with a resolution of 640 × 512 pixels and a frame rate of 55 Hz was used to record the images. Calibration procedures were performed before each test to maintain consistency in the experimental conditions.

Pulsed thermography has been widely applied in various industries, including aerospace, automotive, and composite materials inspection. It is particularly effective for detecting internal defects in carbon fiber-reinforced polymers (CFRPs) and other advanced materials. Recent advancements in signal processing techniques, such as independent component analysis, have further enhanced its capability to detect and quantify defects with improved accuracy and reliability [11,18].

### 2.2. Thermographic Signal Reconstruction—TSR

Thermographic Signal Reconstruction (TSR) is a widely adopted algorithm in the field of infrared thermography. As described by [7], TSR focuses on reducing both the spatial and temporal resolution of thermographic data sequences, significantly streamlining the volume of information to be processed. This approach mitigates common challenges in pulsed thermography by transitioning the data into a logarithmic domain.

TSR is based on Fourier’s law of heat conduction, which describes the thermal diffusion process in a material. To facilitate data analysis, the classical one-dimensional Fourier equation is transformed into a logarithmic domain, which enables the representation of temperature decay in a linearized form. By applying this transformation, the temperature variation over time can be expressed as follows:

(4) $$\ln\left(\Delta T\right)=\ln\left(\frac{Q}{e}\right)-\frac{1}{2}\ln\left(\pi t\right)$$

This logarithmic transformation of Fourier’s one-dimensional solution is adjusted to fit a time series using a polynomial function of degree *n*. This polynomial approximation enables the reconstruction of temperature decay curves for each pixel, allowing the identification of defective regions that deviate from the expected thermal response. The resulting thermographic sequence is then converted into images representing the n+1 polynomial coefficients, facilitating the creation of synthetic thermograms.

(5) $$\ln\left(\Delta T\right)=c_{0}+c_{1}\ln\left(t\right)+c_{2}\ln^{2}\left(t\right)+\cdots +c_{n}\ln^{n}\left(t\right)$$

In Equation (5), ΔT denotes the temperature change over time *t* for each pixel (i,j). This polynomial-based representation transforms the thermographic data into a series of coefficients, from $c_{0}\left(i,j\right)$ to $c_{n}\left(i,j\right)$, which are used to generate images reflecting the temperature variation (i,j,t). These coefficients also allow for the computation of derivatives, accounting for temporal noise and changes.

The TSR method offers notable advantages, including noise reduction, analytical flexibility, and efficient data compression. It also enables the interpolation of temperature values between data acquisition intervals. The first derivative of the polynomial reveals the cooling rate, while the second derivative highlights variations in this rate, providing deeper insights into the thermal behavior of materials [19].

Several alternative preprocessing techniques have been explored in the literature to enhance defect detection in infrared thermography. Principal Component Thermography (PCT) [18] and Independent Component Analysis (ICA) [11] are commonly used to extract key features from thermal sequences by decomposing the data into orthogonal or statistically independent components. While these methods effectively reduce noise and emphasize defect-related patterns, they often require parameter tuning and may suffer from information loss during dimensionality reduction.

In contrast, TSR preserves the original thermal decay behavior by utilizing polynomial approximations, allowing direct interpretation of defect-related variations in thermal signals. The polynomial-based approach enhances defect contrast while maintaining spatial integrity, which is particularly advantageous for integration with deep learning models such as U-Net. Additionally, unlike PCT and ICA, TSR does not rely on statistical assumptions about data distribution, making it more adaptable to different materials and imaging conditions.

However, TSR is not without its challenges. The effectiveness of the polynomial reconstruction depends on the selection of parameters, such as polynomial degree and derivative order, which may require optimization for different defect types. Furthermore, environmental factors, such as temperature fluctuations and reflections, can introduce uncertainties in real-world applications. To mitigate these issues, future research could explore adaptive TSR models that dynamically adjust reconstruction parameters based on material properties and experimental conditions.

Despite these challenges, the results presented in this study demonstrate that TSR preprocessing significantly enhances defect segmentation accuracy. By refining the thermal signal before deep learning-based segmentation, the proposed method improves the robustness and reliability of defect detection in CFRP materials, reinforcing its potential for advancing non-destructive testing techniques [7].

### 2.3. Deep Learning-Based Methods Used in Thermography for NDT

Recent advancements in deep learning have significantly enhanced the classification and segmentation of pulsed thermography (PT) data. For instance, Mask-RCNN has been employed to analyze synthetic PT datasets, effectively detecting abnormal regions in composite materials [20]. Similarly, U-Net models have been applied to segment defect regions in curved CFRP samples inspected via PT, demonstrating strong performance in handling complex geometries [20]. Faster-RCNN architectures, incorporating Inception V2 and Inception ResNet V2, have been utilized to identify defects in composite materials through thermographic images. Comparisons of average precision reveal that the Inception V2-based model achieves superior accuracy compared to the Inception ResNet V2 model [20].

Further research has explored hydrogen-based deep neural networks, which integrate temporal and spatial features to detect defects in composites and coatings [21]. Another study applied machine learning classification techniques to detect impact damage in composite samples using PT, achieving classification accuracies ranging from 78.7% to 93.5% [22]. Recurrent and feed-forward neural networks have been investigated for identifying defects in non-planar CFRP components, with LSTM networks outperforming feed-forward networks in handling temporal dependencies [23]. In addition, neural networks trained on raw thermographic data were compared to those trained on TSR-preprocessed data, with the latter significantly improving segmentation accuracy [24].

Generative adversarial networks (GANs) have also shown promise in processing thermographic data. A GAN-based approach for thermal image enhancement has been developed to improve defect visibility in CFRP components [25]. The IRT-GAN model, trained on six datasets of simulated thermographic data, leverages TSR coefficients as input to produce accurate and segmented images, further demonstrating the potential of GANs in automated defect detection [26].

Expanding on these innovations, a novel approach has been developed by combining DeepLabv3 and BiLSTM models for segmenting thermographic images of CFRP composites. This integration, applied for the first time in infrared imaging, has demonstrated substantial improvements in defect detection accuracy. Experimental comparisons revealed that the DeepLabv3-BiLSTM combination achieved an F1-Score of 0.96 and an IoU of 0.83, surpassing other methods such as U-Net. These findings emphasize the importance of incorporating both temporal and spatial features to analyze complex thermal patterns, thereby advancing the inspection of composite materials through pulsed thermography [27].

Despite significant advancements, the literature persists in a critical gap regarding the integration of TSR as a core preprocessing technique in thermal analysis workflows. This study seeks to address this gap by exploring the impact of TSR on improving the accuracy and reliability of deep neural networks for defect detection in NDT. Additionally, it introduces novel approaches for inspecting high-performance materials like CFRP, advancing the capabilities of existing methodologies.

### 2.4. U-Net

The U-Net neural network has emerged as a cornerstone in image segmentation, extensively utilized in medical imaging and industrial inspection applications [28,29]. Initially proposed by Ronneberger et al. [29], the architecture has gained recognition for its ability to deliver high accuracy even with limited training data. Its dual capability to capture global context and preserve fine-grained localization details has made it indispensable in various domains [28].

**Figure 4:**
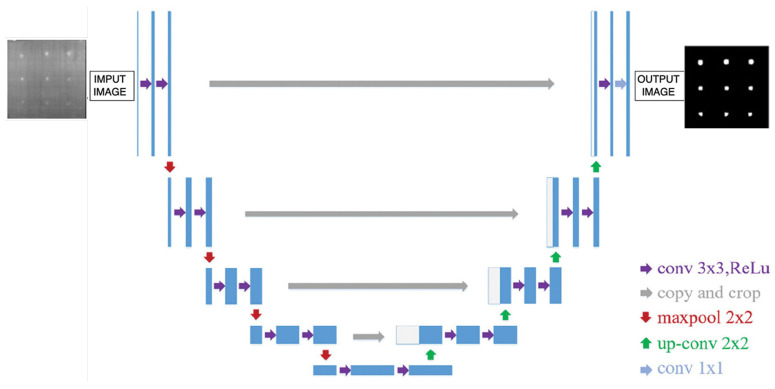
The architecture of the U-Net.

The architecture of U-Net is defined by its symmetric encoder–decoder structure, as depicted in Figure 4. The encoder, often referred to as the contracting path, progressively reduces the spatial resolution of input images while extracting hierarchical features through convolutional and max-pooling layers [29]. This step enables the network to model complex high-level abstractions, which are essential for identifying significant patterns within the data [28].

In parallel, the decoder, or expansive path, reverses this process by gradually restoring the spatial resolution through upsampling and convolutional layers. Crucially, skip connections between corresponding layers in the encoder and decoder allow the network to combine precise localization information from earlier stages with the contextual features learned during downsampling [28,29]. This integration enhances segmentation accuracy by ensuring the alignment of detailed and contextual features.

Beyond its initial application in biomedical imaging, U-Net has been adapted to various industrial tasks, including the detection of defects in materials inspected through infrared thermography [28]. Its resilience to data scarcity and effectiveness in handling diverse segmentation challenges underscore its versatility and enduring importance in modern image analysis pipelines [29].

The implementation of the model was performed using the PyTorch 2.6 library, which enabled efficient processing of large-scale image datasets through GPU acceleration [30]. The model architecture is built from sequential blocks, each consisting of two 2D convolutional layers followed by batch normalization and ReLU activation functions, forming the core computational operations.

The encoder, or contraction path, reduces the spatial resolution of the input features while increasing the depth of representation. This path consists of four levels, each including a double convolutional block and a max-pooling operation. The number of filters increases progressively, starting from 64 and doubling at each level, reaching 512 at the deepest layer. Conversely, the decoder, or expansion path, restores spatial resolution using bilinear upsampling. Each upsampling step is followed by the concatenation of the corresponding feature maps from the encoder and additional convolutional blocks, refining the representations and ensuring accurate segmentation.

The final output is produced by a single convolutional layer with one filter, mapping the refined features into a binary segmentation mask. This output highlights the regions of interest within the input image, enabling precise defect detection.

Optimization of the model was achieved using the Adam optimizer, with an initial learning rate of $10^{-5}$. To enhance the training process, the learning rate was dynamically adjusted using the ReduceLROnPlateau scheduler, which reduces the learning rate when the validation loss stagnates, inspired by adaptive learning rate strategies proposed by [31]. The Binary Cross Entropy with Logits Loss (BCEWithLogitsLoss) function was used to compute the loss. The training was conducted for 60 epochs in a GPU-accelerated environment, ensuring efficient computation and faster convergence.

### 2.5. Training, Validation, and Test Data

The dataset collected in the experimental setup illustrated in Figure 3 comprises 1053 preprocessed thermographic images using the TSR technique. These images were systematically divided into three distinct subsets: training, validation, and testing. A total of 737 images (70%) were allocated for training, ensuring the model could learn effectively from a diverse set of examples. Another 158 images (15%) were set aside for validation, enabling the tuning of hyperparameters and monitoring performance during training. Finally, the remaining 158 images (15%) were designated for testing, ensuring an unbiased evaluation of the model’s segmentation capabilities. This stratified division guarantees that each subset maintains a balanced distribution of defects, contributing to a robust and generalizable model.

To facilitate model training, all collected images were meticulously annotated by an expert. The annotation process was carried out using the VGG Image Annotator (VIA), a web-based tool developed by the Visual Geometry Group at the University of Oxford [32]. The VIA tool was chosen for its user-friendly interface, flexibility, and capability to handle diverse annotation tasks efficiently.

The annotation workflow consisted of four main stages to ensure precision and consistency in defect labeling:

- Image Processing and Standardization: Before annotation, all thermal images were uploaded to the VIA tool and pre-processed to maintain uniform resolution and format across the dataset. This step was crucial to ensure consistency in defect representation.
- Manual Region Marking: An expert manually outlined the Regions of Interest (ROIs) that corresponded to defect locations within the CFRP material. These delineations served as the basis for accurate defect segmentation.
- Defect Classification: Each annotated ROI was assigned a specific label to categorize the defect type. This structured labeling approach helped distinguish different types of defects clearly.
- Validation and Quality Assurance: To ensure annotation reliability, a cross-validation process was conducted involving multiple annotators. Any inconsistencies were resolved through discussions, and necessary adjustments were made to maintain a high standard of accuracy.

**Figure 5:**
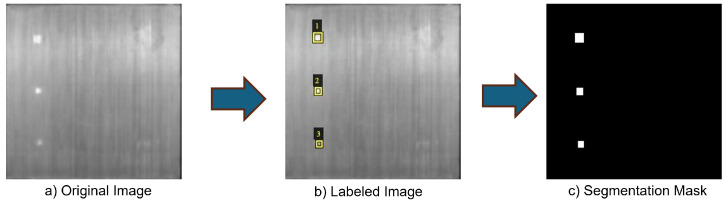
Visual comparison of the manually annotated image and the generated segmentation mask.

The involvement of multiple annotators and the previous knowledge of the positions of the polyimide film tapes prior to molding ensured that all defects were correctly labeled. Once the annotation process was finalized, the labeled regions were compiled into a ground truth dataset, which served as a reference for training and validating the segmentation models. The creation of precise ground truth masks was essential for aligning model predictions with actual defect structures, thereby improving the reliability of the defect detection system. Figure 5 illustrates a visual comparison of the original thermal image, the annotated regions, and the corresponding segmentation mask, showcasing the transformation of raw data into a structured dataset optimized for deep learning applications.

### 2.6. Evaluation Metrics

To evaluate the efficiency of the data processing approach in defect detection, both qualitative and quantitative analyses were conducted. The qualitative analysis involved comparing the processed images step-by-step to observe visual improvements. Quantitatively, metrics such as the signal-to-noise ratio (SNR) were used to compare the original images with those enhanced by the TSR technique. Additionally, segmentation performance was assessed using metrics like the F1-Score and Intersection over Union (IoU).

The SNR measures the contrast between defective regions and surrounding intact areas, offering a dynamic range representation. For each defect, a region within the defect and a noise region representing the defect-free area are selected. In this study, SNR values are calculated using Equation (6), following the methodology described by [33].

(6) $$SNR=\frac{S}{N}=20\cdot \log_{10}\left(\frac{\mathrm{abs}(S_{\mathrm{area}(\mathrm{mean})}-N_{\mathrm{area}(\mathrm{mean})})}{\sigma }\right)\left[\mathrm{dB}\right]$$

In Equation (6), $N_{\mathrm{area}(\mathrm{mean})}$ represents the average pixel intensity within the defective region, $S_{\mathrm{area}(\mathrm{mean})}$ is the average intensity in the defect-free area, and σ is the standard deviation of pixel intensities in the defect-free region.

The F1-Score evaluates model accuracy by balancing precision and recall, making it particularly useful for imbalanced datasets. It is computed using Equation (7), as the harmonic mean of precision and recall [34].

(7) $$F1=2\cdot \frac{\mathrm{Precision}\cdot \mathrm{Recall}}{\mathrm{Precision}+\mathrm{Recall}}$$

*IoU* is another widely used metric for segmentation tasks, quantifying the overlap between the predicted regions and the actual ground truth. It is calculated using Equation (8) by dividing the intersection area by the union area.

(8) $$IoU=\frac{\mathrm{Intersection}\;\mathrm{Area}}{\mathrm{Union}\;\mathrm{Area}}$$

Higher *IoU* values, closer to 1, indicate greater accuracy in identifying defects, while values near 0 suggest poor performance. In this study, *IoU* was adapted to calculate overlaps using defective and non-defective pixels instead of bounding boxes, ensuring the metric’s relevance to thermal image segmentation.

## 3. Results

**Figure 6:**
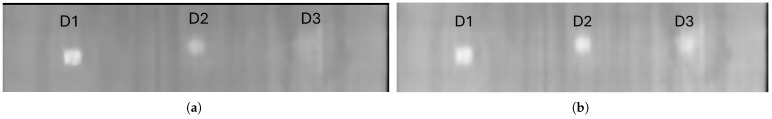
Comparison between images with and without processing. (**a**) Top section of raw data, (**b**) data processed with TSR.

The application of the TSR technique demonstrates a substantial improvement in the visualization of defects within CFRP samples, as shown in Figure 6. The comparison focuses on three distinct defects, labeled D1, D2, and D3, located at varying depths within the material. In Figure 6a, which presents the raw image, D1, the shallowest defect, is moderately visible, while D2 and D3, corresponding to intermediate and deeper defects, respectively, become increasingly difficult to detect due to reduced contrast and the effects of thermal diffusion.

In contrast, Figure 6b, representing the TSR-processed image, reveals a significant enhancement in defect visibility. D1 appears with sharper definition, and both D2 and D3 exhibit noticeably improved contrast, making them easier to identify. The deeper defect, D3, in particular, benefits substantially from TSR processing, as its thermal signature is amplified, compensating for the challenges associated with its depth. These results highlight TSR’s capability to enhance thermal contrast, improving defect detectability across various depths and enabling more reliable inspection and analysis of composite materials.

**Figure 7:**
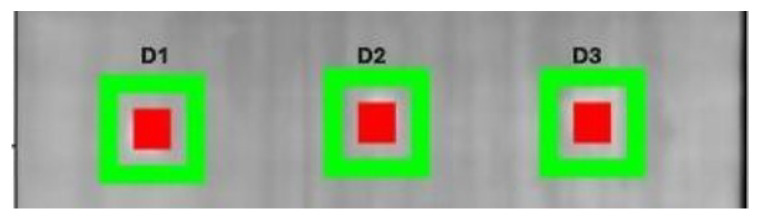
Defect areas (red) and intact areas (green).

The signal-to-noise ratio (SNR) was determined following the methodology described in Equation (6). As shown in Figure 7, defective regions (highlighted in red) and intact regions (highlighted in green) were selected for the SNR calculation. This process was performed on a representative thermal image and subsequently repeated across all frames in the thermal sequence.

The results of the SNR calculations for each identified defect are summarized in Table 3, providing a quantitative assessment based on the defined regions in the image. This approach ensures consistency in evaluating the contrast between defective and defect-free areas, facilitating a detailed analysis of the thermal sequence.

The results presented in Figure 7 and Table 3 illustrate the impact of the TSR technique on improving the Signal-to-Noise Ratio (SNR) for defect detection in CFRP samples. In the raw images, the SNR values for defects D1, D2, and D3 were 14.03 dB, 13.84 dB, and 2.98 dB, respectively, indicating that deeper defects (e.g., D3) are harder to detect due to their weaker thermal signal. After applying the TSR processing, significant improvements in SNR were observed, with D1, D2, and D3 reaching values of 18.41 dB, 15.72 dB, and 17.80 dB, respectively.

This demonstrates the effectiveness of TSR in enhancing the contrast and clarity of defects, particularly for deeper anomalies like D3, which showed a remarkable increase in detectability. The improved SNR values reflect the enhanced ability to distinguish defective regions from intact areas, confirming TSR’s capability to process thermal signals effectively and provide more reliable insights for defect identification in materials with varying depths. These results underscore the importance of incorporating advanced preprocessing techniques in thermographic analyses to address challenges related to signal attenuation and noise.

To evaluate the segmentation performance, the processed data were utilized as input to the U-Net architecture, aiming to delineate the defective regions within the thermal images. The U-Net model underwent two distinct training and validation phases to enable a comparative analysis of its performance. In the initial phase, the network was trained using raw thermal images acquired directly from the experimental setup, without any preprocessing. In the subsequent phase, the same model was trained and validated using thermal images enhanced through the TSR technique.

**Figure 8:**
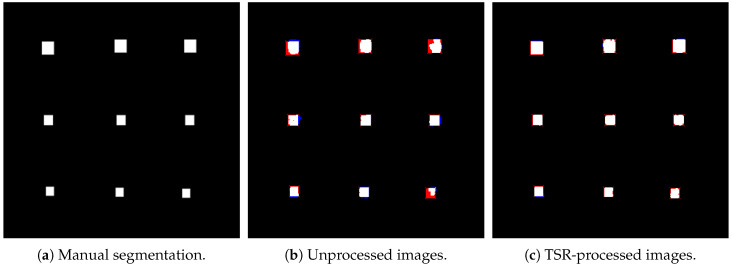
Comparison of segmentation results. Red pixels indicate false negatives (regions where defects were present but not detected), while blue pixels indicate false positives (regions where defects were incorrectly detected). (**a**) Ground truth segmentation, (**b**) segmentation from unprocessed images, (**c**) segmentation from TSR-processed images.

Figure 8 showcases the results of this comparison, displaying three sets of segmentation outputs for all nine defects: (a) ground truth segmentation manually annotated by an expert; (b) the predicted segmentation produced by the U-Net model trained with raw thermal data; and (c) the segmentation results obtained when the model was trained with TSR-preprocessed images. In these images, red pixels indicate regions where the model failed to detect defects (false negatives), while blue pixels represent areas where the model incorrectly classified non-defective regions as defective (false positives). This comparison highlights the influence of preprocessing on the network’s ability to accurately segment and identify defective areas across varying depths within the material.

The results presented in Figure 8 provide a direct visual comparison of segmentation performance under three conditions: manual annotation, model predictions using raw thermal images, and predictions with TSR-processed images. The manual segmentation (a) serves as the ideal reference, outlining the precise defect locations. When comparing (b) and (c), a higher density of red and blue pixels is observed in the segmentation from raw thermal data, indicating greater difficulty in distinguishing defective from non-defective regions. This effect is particularly pronounced in deeper defects (rightmost column), where low thermal contrast leads to misclassification. However, the segmentation results from TSR-processed images (c) show a notable reduction in false positives and false negatives, demonstrating that preprocessing significantly improves model performance. These results reinforce the effectiveness of TSR in enhancing defect segmentation accuracy.

**Figure 9:**
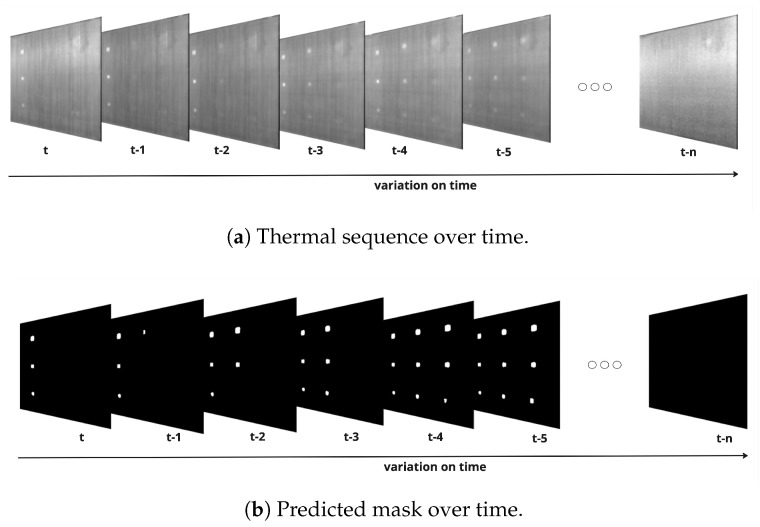
Comparison of original and predicted images over time [27].

To assess the segmentation performance quantitatively, thermal image sequences were processed by the models, as shown in Figure 9. Each frame in the sequence was segmented, and evaluation metrics, including F1-Score and IoU, were calculated for every segmented frame. These metrics provide a frame-by-frame assessment of how well the models identify defective regions. Subsequently, the average values of these metrics across the entire sequence were computed to summarize the overall performance of the models.

Figure 9 illustrates the original thermal image sequence and the corresponding segmented results. Panel (a) depicts the original thermal frames over time, capturing the evolution of heat distribution across the material. Panel (b) shows the predicted segmentation outputs, highlighting the regions identified as defects. This comparison enables a comprehensive evaluation of the model’s ability to accurately segment defects at different depths and time frames.

The quantitative results are summarized in Table 4, which compares the U-Net model trained on TSR-processed data with models previously reported in the literature, including U-Net and DeepLabv3+BILSTM from [27]. This comparative analysis underscores the impact of TSR preprocessing on model accuracy and demonstrates the advantages of advanced architectures in capturing temporal and spatial features effectively.

The results presented in Table 4 highlight the impact of preprocessing and model architecture on segmentation performance. The U-Net model, when trained without TSR preprocessing, achieved an F1-Score of 0.9142 and an IoU of 0.6141, demonstrating its baseline capability in segmenting defects. However, the DeepLabv3+BILSTM model, even without TSR preprocessing, outperformed the U-Net with an F1-Score of 0.9629 and an IoU of 0.8312, likely due to its ability to incorporate temporal and spatial features. When TSR preprocessing was applied to the U-Net model, there was a significant improvement, with an F1-Score of 0.9903 and an IoU of 0.9516.

This demonstrates the critical role of TSR in enhancing thermal image quality and segmentation accuracy. Notably, the U-Net model with TSR preprocessing surpassed the DeepLabv3+BILSTM model without TSR, emphasizing the importance of preprocessing techniques in improving segmentation performance. Despite these benefits, TSR preprocessing introduces an additional computational cost compared to raw image processing. The polynomial fitting and derivative calculations required for TSR increase both memory usage and processing time. However, the impact remains manageable for most applications, as the preprocessing stage is performed offline before model inference. For real-time scenarios, optimization strategies such as parallel processing and hardware acceleration could be explored to minimize computational overhead. These findings underscore the value of combining advanced preprocessing methods like TSR with neural networks to achieve superior results in defect detection and segmentation tasks.

## 4. Conclusions

The findings of this study have direct implications for the detection of CFRP defects in industrial applications. By leveraging TSR preprocessing, the accuracy and reliability of defect segmentation in pulsed thermography can be significantly enhanced. The ability to amplify defect visibility, particularly in low signal-to-noise ratio (SNR) conditions, suggests that this methodology could be effectively deployed in aerospace, automotive, and infrastructure industries where CFRP integrity is critical. Improved segmentation accuracy translates into more reliable non-destructive testing (NDT) procedures, reducing the likelihood of undetected structural damage and enabling predictive maintenance strategies.

This study demonstrated the critical role of thermographic signal reconstruction (TSR) using polynomial approximations in enhancing the defect detection and segmentation of CFRP laminates through pulsed thermography. By preprocessing thermal images with TSR, significant improvements were observed across all evaluation metrics, highlighting its ability to enhance the signal-to-noise ratio (SNR) and improve the overall quality of input data for neural networks. The most notable SNR improvements were observed in defect D4, which increased from 9.33 dB to 22.36 dB, and defect D9, which improved from −1.48 dB to 9.02 dB, illustrating the technique’s effectiveness in amplifying defect visibility and contrast.

The U-Net model trained with TSR-processed images achieved exceptional results, with an IoU of 95.16% and an F1-Score of 99.03%, far outperforming the unprocessed model, which achieved an IoU of 61.41% and an F1-Score of 91.42%. These findings underscore the importance of preprocessing techniques in non-destructive testing workflows, enabling more precise and reliable segmentation of defects, particularly for deeper anomalies that are traditionally challenging to detect. Furthermore, the TSR-preprocessed U-Net model achieved comparable, and in some cases superior, performance to more complex architectures like DeepLabv3+BILSTM.

Despite these advancements, some limitations should be acknowledged. TSR preprocessing introduces an additional computational cost due to the polynomial fitting and derivative calculations, which increase processing time and memory usage. While this is manageable for offline analysis, further optimizations would be necessary for real-time applications. Additionally, the selection of TSR parameters, such as polynomial degree, has a direct impact on segmentation performance and may require tuning for different material properties and defect characteristics. Another limitation is that this study was conducted under controlled experimental conditions. To ensure broader applicability, future work should validate the approach across a wider range of CFRP structures, defect types, and real-world environmental conditions.

Future research could explore the integration of TSR with complementary preprocessing strategies, such as wavelet transforms or feature extraction techniques, to further enhance defect segmentation. Additionally, the application of Transformer-based models and hybrid deep learning approaches could improve the accuracy and generalization capability of segmentation networks. Another promising avenue is the optimization of computational efficiency, either through parallel processing techniques or the development of lightweight neural architectures, making TSR preprocessing viable for real-time inspections. Finally, expanding this study to include validation in industrial settings, such as automated CFRP defect detection in aerospace maintenance, could bridge the gap between research and practical deployment.

This research highlights the value of integrating advanced preprocessing methods with neural networks, providing a robust framework for defect detection in high-performance materials such as CFRP laminates. The combination of TSR with deep learning-based segmentation models significantly improves defect detection accuracy and robustness, demonstrating its potential for practical applications in industrial non-destructive testing. Further developments in this field could lead to more efficient and scalable defect detection systems, contributing to safer and more reliable composite material inspections.

## Figures and Tables

**Table 1:** Thermal properties of APC-2/AS4 [14].

Property	Value	Unit
*k*: Thermal conductivity (Longitudinal)	5.65	W/mK
*k*: Thermal conductivity (Transverse)	0.35	W/mK
$C_{p}$: Specific heat ^(*a*)^	1310	J/kgK
ρ: Density	1584	kg/m³

^(*a*)^ at constant pressure.

**Table 2:** Mechanical properties of APC-2/AS4 [15].

Property	VAlue	Unit
Tensile Properties		
Longitudinal Tensile Modulus	127.6	GPa
Transverse Tensile Modulus	10.3	GPa
Longitudinal Tensile Strength	2132	MPa
Transverse Tensile Strength	95.2	MPa
Shear Properties		
In-Plane Shear Strength	82	MPa
Poisson’s Ratio		
Longitudinal	0.32	
Transverse	0.022	
Thermal Property		
Glass Transition Temperature	143	°C

**Table 3:** Calculation of SNR in decibels (dB).

Defects	SNR Original (dB)	SNR TSR (dB)
D1	14.03	18.41
D2	13.84	15.72
D3	2.98	17.80

**Table 4:** Comparison of segmentation metrics for different models.

Model	Preprocessing	F1-Score	IoU
U-Net *	Without TSR	0.9142	0.6141
DeepLabv3+BILSTM *	Without TSR	0.9629	0.8312
U-Net	With TSR	0.9903	0.9516

* Adapted from [27].

## Data Availability

The dataset in this study is available at: Garcia Vargas, Iago; Fernandes, Henrique (2025), “Thermal Inspection Dataset for Defect Segmentation in CFRP Laminates”, Mendeley Data, V1, doi:10.17632/jrsb4b9yy5.1.
